# Lectures on the Nature and Treatment of Deformities, Delivered at the Royal Orthopædic Hospital, Bloomsbury Square, (London)

**Published:** 1846-06

**Authors:** 


### ARTICLE VI.

*Lectures on the Nature and Treatment of Deformities*, delivered at the Royal Orthopædic Hospital, Bloomsbury Square, (London.) By R. W. Tamplin, F. R. C. S. E., Surgeon to the Hospital. With numerous illustrations. Philadelphia: Ed. Barrington and George D. Haswell. 1846. pp. 216. 8 vo. (From the Publishers.)

While systematic works of Surgery abound, and most important surgical subjects have been well and fully treated in monographs, a treatise on the subject of deformities has been a desideratum in English Medical literature. This is doubtless owing to the fact, that until quite recently, these affections have not been considered as coming within the pale of surgery—they have even been ranked with mal-formations, and the subjects of them have been subjected to an absurd popular prejudice, which excluded them from many of the privileges and enjoyments of their fellow men; in the same manner as by the mosaic law, they were excluded from the dignity of the priesthood. Orthopædic Surgery is not only one of the newest, but also one of the brightest triumphs of science, and may teach us not to despair of the discovery of means of cure for those diseases at present deemed most hopeless.

The Orthopædic Institution in Bloomsbury Square, owes its origin, as is stated, "to the benevolent exertions of Mr. Quarles Harris, who, having experienced relief in his own family, devoted himself to the formation and support of this Charity, for the benevolent purpose of extending the same benefits to the poor, which he knew the rich could command."

It is our purpose to give an analysis of a part of the work of Mr. Tamplin, which is entirely practical in its character, without criticising his opinions. We would, however, say in the commencement, that the style of the book is, in many parts, loose and incorrect, that the author's views of the pathology of the diseases producing deformities, seem far from profound. But on the other hand it may be said with equal truth, that the work is very modest in its pretensions, that its practical views are generally correct, and always on the side of moderation and safety, and that all those extreme and violent measures and operations that have discredited this branch of surgery in the public mind, are here discountenanced.

The following extracts will show our author's views on the subject of the division of tendons, their re-union, and the period at which extension should be made. On this latter point our experience has shown us that in a great number of cases, extension may be applied with perfect safety immediately after the division of the tendon, but it is by no means certain that this is universally true, and when much or long-continued force is required, this should not be practised.

"The principles laid down by Delpech, in his Orthomorphie, published at Paris in 1828, are comprised in the following rules:—

"1st. 'A tendon to be divided must not be exposed; and its division should be made by turning the instrument on one side, so that the line of the incision may not be parallel to the division of the skin; without this precaution risk of exfoilation of the tendon is incurred.'

"2d. 'Immediately after division of the tendon, the divided ends should be brought into contact with each other, and kept in this position by a suitable apparatus during the entire period necessary for their union.'

"3d. 'Inasmuch as it can only take place by the intervention of an intermediate fibrous substance, this substance, before it has become firm, can, and should be, extended gradually and carefully, until it has assumed a degree of length equal to the shortened muscle.'

"4th. 'When this degree of extension has been effected, the parts should always be fixed in the position, and kept so until the new substance has acquired its requisite degree of consolidation.'

"These, gentlemen, are the principles upon which we are now acting, and from which we depart but in the slightest degree; they embody the entire doctrine of the treatment of deformity, and have only to be followed out carefully to insure success. There can be no question that had Delpech been spared to enjoy the common number of years allotted to man, he would have extended to every variety of deformity, the new views which he so graphically announced. It was not only in deformity in the feet, and the physiology of the division of tendons, that Delpech shone so conspicuously, but also on lateral curvature of the spine, and on dislocations of all kinds, on which subjects his works must be considered as second to none in originality and in correctness of principle. It is gratifying to find, that Stromeyer, who has been fortunate enough to extend the treatment of the distinguished surgeon of Montpelier, has generously acknowledged the merits of Delpech; for he says, 'Although the division of tendons has been recommended long since as a mode of removing certain contractions, yet the credit of having set this operation on a proper scientific basis undoubtedly belongs to Delpech, inasmuch as he showed the peculiar advantages derived from a new fibrous substance between the divided ends of the tendon, thus giving to this method of operating, its true practical value, and enabling us to avoid rendering inactive the muscles whose tendons have been divided:' an acknowledgment on the part of Stromeyer which does equal credit to his head and heart. In his work 'On Operative Orthopædic Surgery,' Stromeyer observes—'Delpech laid it down as a rule, that the surgeon should encourage the formation of sufficient substance between the two divided ends of the tendon to maintain the function of the muscle, and should not destroy the new union by immediate extension, but commence extension some days after the operation: this rule is most important for the safe performance of orthopædic operations, and its value should be duly estimated. The idea that the elongation of the muscle is effected through the cicatrix, is a false one; the extent of the substance or cicatrix, is quite inadequate for this purpose. In some cases of pes equinus the gastroenemii are two or three inches too short; and, in wry neck, the sterno-mastoid is equally short, yet the cicatrix, after the cure, is but a few lines long. The elongation of the muscle must be effected in these cases at the cost of its contractility, and thus the incision of its tendon acts not only on its mechanical, but also on its vital properties, and, by the temporary dimінution of its irritability, its contractile power is diminished, and any increase of it prevented. This view is confirmed by observations made in cases where the tendons of sound muscles have been lacerated. The following is an instance:—A medical man, of lax fibre, ruptured his tendo-Achilles seven years ago, and, in spite of the injury, walked about a few days afterwards; at the present time he walks about, dragging his leg after him, like a paralytic man, although the cicatrix is only a few lines long, and would be only considered as likely to produce any lameness in those motions which require forcible contraction of the calf. The injury and subsequent want of use have evidently here caused a loss of power in the calf, which, in his feeble condition, could not readily be restored. It is a remarkable circumstance, that when the tendo-Achilles unites in an imperfect manner after injury, the foot is not drawn up by the flexor muscles, but hangs like a loosely connected part, showing that the diminished irritability of such a mass as the calf of the leg exerts a weakening influence on the entire extremity. That any person should commence extension immediately after the operation, and attempt to restore the limb to its natural position, using, like Sartorious, a degree of violence that makes us shudder to think of, is neither necessary nor advisable.'

"The immediate restoration of a limb in its natural position is not to be recommended, for by extension before the healing of the wound in the skin, the parts are liable to inflame or suppurate; by gradual extension, the contractility of the muscle, the tendons of which have been divided, is interrupted for a time, and restored by the stretching and motion of the parts when the foot is again used. 'In all my cases,' adds Stromeyer, 'of division of the tendo-Achilles, the use of the muscles of the calf has been completely restored. Dr. Weiss informs me that at Paris the use of the muscles of the calf is not always restored. This may probably depend on the immediate separation of the ends of the tendon, which does not prevent its healing, but hinders the restoration of the function of the part; it is also possible that, by the extension of the intervening substance, which, from immediate extension, is very thin, the cicatrix itself may be lacerated.'

* * * "I have had two opportunities of examining the condition of tendon that had been operated upon during life, after death; the one was a boy about seven or eight years of age, in whom the tendo-Achilles was divided for complete talipes equinus, the heel being elevated to its full extent: he was of weak, delicate, and unhealthy constitution, and the foot was brought into position three or four weeks after the operation; the uniting medium was at that time nearly two inches in length, soft, yielding, and exceedingly weak. It gradually, however, strengthened, contracted upon itself, and became as strong, to all appearance, as the original tendon; nor could any irregularity or thickening be detected. Twelve months after the operation he was attacked with scarlet fever, of which he died. His father informed me of it, and offered to allow an examination. On examining it externally, no trace of its having been divided could be detected; it possessed the same prominent uninterrupted outline as its fellow; the point of puncture could with difficulty be detected. On removing the skin and cellular tissue, there was no evidence of a wound having been inflicted, no adhesions, thickening, or swelling, and on laying open the sheath the tendon presented one uniform and natural appearance; so much so, that one was almost led to doubt the possibility of its having been divided. I then made a longitudinal section, but could discover no alteration with the naked eye, except a sort of globular appearance in one spot, but not sufficiently differing from the tendon itself to make us positive. The other was a congenital case of talipes varus, in a child of eight months of age when operated upon, and who died from hooping-cough and head affection eight months after the operation. The tendo-Achilles, the anterior and posterior tibial tendons, had been divided. The same perfection existed here, and at the examination, no trace of any kind could be detected, in appearance or by sense of touch.

* * * "Von Ammon (*De Physiologiâ Tenotomiæ*) gives the following account of the union of tendons:—'When a tendon is divided a slight degree of pain occurs, but no spasm of the part. In a short time a gap is produced by the contraction of the divided tendon, the principal part of the contraction taking place in the part of the tendon above the division.' This gap is soon filled up with blood, which he says, chiefly flows from the upper end of the divided tendon. This blood soon coagulates, and in this process unites firmly with the surrounding parts, and more especially with the wounded surfaces of the tendon, the ends of the tendon presenting at this time an appearance as if they had been tied round with a thread. The next change consists in the effusion of coagulable lymph beneath the effused blood, from the surrounding tendon and adjacent parts; this lymph becoming soon marked with conical and thread-like streaks of a white colour, which extend from the two divided ends of the tendon, and seem to shade gradually into each other. This soft substance thus thrown out, instead of remaining as a pulpy semi-transparent mass, soon becomes connected into a structure which resembles, to a certain degree, the structure of tendon. This substance is not, however, true tendon in structure, although it exercises the same functions: on the surface and in smoothness it resembles tendon; but it differs from it in presenting, in its early stage, a substance of an udder colour, and of a more compact form, and afterwards presenting a more blue colour than real tendon. The motions of this new substance are more confined than real tendon, partly on account of its want of elasticity, and also from its adhesion to the surrounding parts. This new substance is formed in about fourteen days."

Our author commences with deformities of the feet, "the treatment of these being the groundwork of the treatment of deformities in general," and then proceeds to those of the knee, hip, spine, neck and upper extremities.

Talipes (club foot) is of four kinds.

1. Talipes equinus in which there is simple elevation of the heel unaccompanied by lateral deformity.

2. Talipes varus in which the heel is elevated, the foot shortened, inverted, semi-rotated and abducted.

3. Talipes valgus or flat-foot, the reverse of the last in which there is no trace of the arch left.

4. Talipes calcaneus the reverse of the first variety, in which the posterior extremity of the os calcis rests upon the ground in walking.

There are in addition to these, cases in which two of the above varieties are combined, as T. equino-varus and T. equino-valgus.

Most of these may be congenital, or be produced after birth. The congenital varieties our author attributes, but probably without sufficient evidence, to the position of the child in the womb.

The non-congenital is produced by a great variety of causes, as paralysis, spasm, inflammation, and a peculiar condition of the nervous centres, inducing gradual contraction, &c.

In whatever manner produced, the following is the condition of the parts in all the different kinds of club foot. The form of the bones is natural. The ligaments are changed, some being lengthened, others shortened. Of the muscles some are contracted, others lengthened, and as a consequence of want of action, both are atrophied. From the same cause there is also diminished circulation, and loss of temperature of the limb. In a recent case which we have seen dissected, there was conversion of the muscular tissue into a fatty and fibrous mass.

"The *treatment*, which we now come to consider, resolves itself into the mechanical solely, or surgical and mechanical combined. With regard to the mechanical, I think sufficient evidence is daily before us of its general failure, from the results witnessed of patients who have been subjected to stretching and rubbing, and the wearing of instruments all their lives; and the existence of this Institution is an evidence of its general utility. I have not much, therefore to say upon this head. It will undoubtedly reduce the severity of the appearance of the deformity, and may, in the slightest amount of contraction, perhaps effect a cure,—at least, we are told it does so. I can only say, I have tried both in congenital and also in non-congenital cases, when the deformity has been slight, and the contraction of the muscle very limited, but I cannot bear testimony to its success on the one hand, or the propriety of it on the other."

Ample experience has convinced us of the justice of these observations, and also that in infants, the sooner the operation is performed the better. The muscles, whose tendons should be divided, will vary in every case. In T. equinus it will be simply the tendo-Achilles; in T. varus that tendon with the anterior and posterior tibial, and the plantar fascia; in the T. valgus, the tendons of the peronei muscles and those of the long extensors of the toes; in T. calcaneus, the tendons of the peroneus tertius, extensor longus digitorum, extensor proprius pollicis, and tibialis anticus. In general it may be stated that those tendons much contracted, should be divided, and this may be judged of by their tension.

The rules for the subcutaneous division of tendons in all parts of the body are nearly the same, viz: a small puncture should be made, about one inch from the tendon to be divided, with a lancet or sharp pointed knife,—the tendon should be divided with a narrow knife, from within outward, or from without inward, as may be most safe or convenient, care being taken to avoid the vessels and nerves. The wound is to be closed with a piece of adhesive plaster. In general, the tendon should be rendered tense at the time of division, and the position of the patient should be that which is most convenient.

We have already given the views of our author in reference to the time proper for applying mechanical extension. This constitutes, in fact, the most important part of the treatment. Many kinds of apparatus have been devised for the purpose; of which our author prefers Stromeyer's foot board or Scarpa's shoe. Whatever instrument is used, it must be applied in such a manner as to make uniform, gentle pressure only, which must be continued for a length of time, varying from two to twelve months. The foot having been restored to its normal position, a boot of some firmness to retain it there is required.

Such is an outline of the plan of treatment recommended by our author, and with some modifications almost universally adopted by surgeons. Readers who have not had experience in the treatment of club-foot, will, of course, be desirous of knowing whether it is likely, in all cases, to be crowned with success. Having had occasion to see treatment applied in several large establishments, as well as in private practice, upon cases of different degrees of severity, and in subjects of various ages, we are of opinion, that in persons of adult age, that are affected with the severer forms of this deformity, especially of the T. varus, no efforts should be made to remove it by treatment. The long continued pressure is exceedingly irksome and painful, and when at length the object is effected, the muscles have so little power, that the patient does not walk as well as before. Our author does not notice this result as occurring in his own practice, but attributes it to extension immediately after the division of the tendons; it is not, however, by any means confined to such cases. When the subject is younger, or the disease more slight, when the patient is docile, and the surgeon's care is constant, the result will be in the highest degree satisfactory.

The pathology of club-foot is that of a great number of deformities—contraction of tendons and muscles, pain, spasm, inflammation, paralysis of their antagonists, &c., are the most common. The treatment also, is upon the same principle. Division of the tendons, where required, extension till a cure is effected, and a suitable support used afterward. These are principal means. The division of the tendons is, however, more useful in the club-foot, than in almost any species of deformity.

Mr. Tamplin next proceeds to consider some cases of deformity attended with paralysis.

"With regard to the non-congenital deformities of the feet, you have, as I have had occasion frequently to observe, paralysis of one or more muscles, and a spasmodic affection of one or more of them; occasionally of the whole voluntary muscular system. When it is combined with paralysis, I have also stated that no known remedy has been discovered, if it has been of long standing, and that we can only remove the deformity, and rest satisfied with artificial support; that the limb is always in an atrophied condition, and possesses the lifeless flabby state which is so peculiar to the paralytic condition; and as we know but little of the pathology of the nerves, I shall not waste either your time or my own by speculation or theory. Suffice it to say, that paralysis exists, and occasionally of both lower extremities, with contraction of one or more muscles, producing either of the deformities before mentioned; frequently varus of one foot, valgus or equino-valgus, of the other; the patient possessing the smallest possible amount of motion in the toes. And of course, if complete paralysis exists, you have no contraction of the knees, the limb lying in any position in which it may be placed, and appearing more like a foreign body than the living extremity, and with the exception of the ligaments by which it is alone held, perfectly passive; if not complete, the knees will also be found contracted, although there may be no available motion. You will occasionally find slight motion in one or other of the extensors of the leg, but not sufficient to be of the slightest use. There is always, however, as far as my present experience goes, more or less available power in the flexors and extensors of the thigh, and from this fortunate circumstance you will be enabled, after you have removed deformity or contraction of the feet, to place the patient in a much better condition—I may say happier condition—compared to that which he has been previously obliged to endure."

Those produced and attended by spasm are even less amenable to treatment than those resulting from paralysis.

"The spasmodic contractions are the most painful and difficult to treat, for although the deformity and contraction may be removed, yet we have hitherto been, and are at present, ignorant of any means of remedying the spasmodic condition of the muscles; and although by division of the tendons, and during the time the uniting medium is soft and yielding, you can easily hold the foot in any position, yet the cause exists, and the patient is unable to control his muscles. As soon as the uniting medium becomes consolidated, the same irregular action is brought into operation, and support the limb by any method you may please to adopt, you cannot remove the cause or the effect. You will, however, even here, improve the condition of the limb with great care; but I would never advise recourse being had to the operation, if the foot or other articulation can be brought into position by the efforts of the hand alone; but if by such continued efforts you are enabled to restore the natural position of the foot, then divide the muscles, which, notwithstanding their spasmodic state, are contracted; and after the removal of the contraction, support the limb, and keep it as much as possible in a fixed position. I know of no cases that are more troublesome than these. It is a curious fact, that in these cases, where every muscle is affected, those of speech and deglutition as well, the intellects are perfect, although apparently weak, as the cause must exist in the brain or its membranes as well as in the spinal cord itself. The involuntary muscles are not in the slightest degree affected. The cause assigned as in most other non-congenital cases, is generally dentition or cerebral irritation, and you will frequently find talipes valgus of the one foot, varus of the other; never, however, in their moie severe forms, as the opponent muscles, although possessing less power, and thereby admitting of the malposition are also in an active spasmodic state, and prevent the foot assuming the more severe malposition. This condition is said to be congenital. I have never seen a case in an infant, and although the parents assert that such is the case, I shall not be satisfied until I see it, as I think it most improbable, except in hydrocephalic congenital states; but even here I have not yet seen it. In the paralytic also you will find one foot affected with talipes varus, the other with valgus, or calcaneo-valgus; in fact, in either of the deformities I have mentioned, you will, in the spasmodic cases, which affect the whole of the muscles of the body, find contraction, either permanent or temporary, of the knees, and if not permanent, they will invariably be found in the flexed position on any attempt to exercise them on the part of the patient. The thighs will also be found adducted, and occasionally more or less contracted, in the flexed position; the pronators and flexors of the hand and arm preponderating in power, so that the patient can exercise no steady well-directed movement, nor even continue the position in which the hand or leg may happen to be placed; as for instance, in the attempt to hold anything in the hand, after having grasped the object, the hand will suddenly open with an irresistable impulse; nor has the patient any power to prevent this occurring.

"It is clear, therefore, gentlemen, that we are in total ignorance of any complete and successful remedy, for these cases; it is therefore useless to enter more into detail, as anything that could be further advanced would be mere speculative theory. Galvanism has, in some cases of the less severe kind, been attended with partial success; but I much doubt if any positive beneficial results have eventually been obtained. You can, therefore, only place the joints in the relative position, and keep them in that position constantly, if the feelings of the patient will admit of it, but occasionally, from the violent spasmodic action of the muscles, you will find it necessary to intermit the treatment."

It is important that this opinion of so competent a judge should not be forgotten, since cases have been published as having been greatly benefitted by treatment. We have ourselves made trial of it under circumstances favorable for testing its merits, but without permanent success. A young man, 16 years of age, had been affected from infancy with spasmodic contraction of all the voluntary muscles, including those of speech. All the members were in a state of rigidity, in a position between flexion and extension, thighs adducted, total inability to raise or feed himself. The intellect apparantly good. He had early been treated by mechanical means alone without success. We divided, first, the tendons of the gracilis and adductor longus muscles, on each side, and applied extension by means of a suitable screw, and in a few days the knees were separated 18 inches, and could be retained there by the patient. Encouraged by this result, we next proceeded to divide the tendons of the biceps, flexor-cruris, semi-tendinosus, and semi-membranosus muscles, the knees were soon straightened, but in the mean time, spasmodic action returned in the adductors, and the knees were approximated as before. This proved the impossibility of success, although no doubt temporary improvement of his condition might have been effected.

*Genu Valgum*, or knock knee is the next subject of consideration.

"It consists in a relaxation and elongation of the internal lateral ligament, and the crucial ligaments must also yield more or less; for in the healthy or normal condition of the joint these ligaments admit of the smallest possible amount of lateral motion. This appears to be the primary condition; and if you will direct your attention to the position of the joint, and observe that it is at a distance from the point or points of pressure in walking or standing, you will easily perceive that if the superincumbent weight of the body is thrown in any but a perfectly straight direction, with regard to the axis of the joint, if the tibia on which the femur rests is in an oblique lateral direction instead of a perfectly horizontal one, corresponding with the articular surfaces of the condyles of the femur, that this bone must press, in walking or standing, in an indirect and abnormal position, producing thus mechanically an increase of the deformity, of whatever kind it may chance to be; and when the foot is felt to incline outwardly, and the knees commence to touch each other, the malposition, as a rule, becomes daily increased in proportion to the extent at which the feet are separated from each other; the greater the distance according to a principle in mathematics, the more powerful will be the effects of pressure occasioned by the weight of the body, and the more rapid the increase of the deformity. The deformity is non-congenital—at least, I have never seen a congenital case, nor can I imagine how it could occur, provided we admit that congenital distortions arise from malposition in utero. The general cause is debility, and the specific causes the effects of detention, the various eruptive diseases to which all children are liable, also hooping-cough (in children)."

Sometimes it is combined with outward inclination of the other knee joint, and is often complicated with secondary affections, such as distortions of the feet, spine, &c. There is usually existing with it impaired digestion, tumid abdomen, flabby flesh, pallor of the skin, &c.

The treatment in the first stage consists in tonic and alterative medicines addressed to the general system, and straight board splints applied to the outside of the leg and thigh. In a more advanced stage, an angular splint with a screw may be necessary, and when tendons are contracted, our author recommends their division—this is not often necessary.

An affection, the opposite of that just described, viz: outward inclination, is sometimes found with the same state of the general system, i. e.: incipient rachitis.

"The treatment is general and mechanical: in children affected with this deformity, you will find very generally that unhealthy state of constitution I have already described, existing with those affected with the last mentioned deformity; this must, of course, be attended to by administering alteratives and tonics, with attention to the diet. The mechanical means we adopt consist of straight splints on the inner side of the leg, extending high up above the knee, and below the internal malleolus, well padded at the points of pressure, and webbing straps applied 'round the leg and splint, so that a constant steady pressure may be kept up: it is only by the most gradual and uninterrupted treatment that good can be obtained, for you must recollect that not only the knees, but the bones also, are affected, and a child cannot bear any amount of continued pressure; it must be so applied that the child is subjected to no pain."

Flexion of the knee, and the various causes which produce it are next considered. There is nothing especially worthy of notice in this chapter, if we except a passage which has been quoted in another place, in reference to the cure of "white swellings." These flexions, in all cases, except in perfect anchylosis, he treats by division of the tendons of the biceps, and semi-tendinosus, and semi-membranosus muscles, and the application of an angular apparatus furnished with a screw. It is our conviction, that the division of the muscles in these cases is entirely unnecessary, and that the extension can be effected with great facility without it.

In speaking of true anchylosis of the knee joint, he pronounces a condemnation of the operation of Dr. Barton, of sawing out a wedge shaped piece from the femur, in order to straighten it. It is sufficient to say of this operation, that it is in these cases the only remedy except amputation, that it has succeeded in all cases in which it has yet been tried, and has as yet, we believe, proved fatal in none.

We have thus given an analysis of the first half of this work, enough, we think, to give our readers a knowledge of its scope and character, certainly all which our time and space will at present allow. The remaining chapters are devoted to the consideration of contraction of the thighs, rachitis, angular, posterior, and lateral curvature of the spine, wry neck, contraction of the lower jaw, of the shoulder joint, elbow, wrists, fingers and toes. In all of these much practical information may be gleaned. D. B.

